# Clinical Outcomes of Cabozantinib in Patients Previously Treated with Atezolizumab/Bevacizumab for Advanced Hepatocellular Carcinoma—Importance of Good Liver Function and Good Performance Status

**DOI:** 10.3390/cancers15112952

**Published:** 2023-05-28

**Authors:** Teiji Kuzuya, Naoto Kawabe, Mizuki Ariga, Eizaburo Ohno, Kohei Funasaka, Mitsuo Nagasaka, Yoshihito Nakagawa, Ryoji Miyahara, Tomoyuki Shibata, Takeshi Takahara, Yutaro Kato, Yoshiki Hirooka

**Affiliations:** 1 Department of Gastroenterology and Hepatology, Fujita Health University, 1-98 Dengakugakubo, Kutsukake-Cho, Toyoake 470-1192, Aichi, Japan; kawabe@fujita-hu.ac.jp (N.K.); a.mizuki0909@gmail.com (M.A.); eizaburo.ono@fujita-hu.ac.jp (E.O.); k-funa@fujita-hu.ac.jp (K.F.); nmitsu@fujita-hu.ac.jp (M.N.); yo-hi@fujita-hu.ac.jp (Y.N.); ryoji.miyahara@fujita-hu.ac.jp (R.M.); shibat03@fujita-hu.ac.jp (T.S.);; 2 Department of Surgery, Fujita Health University, 1-98 Dengakugakubo, Kutsukake-Cho, Toyoake 470-1192, Aichi, Japan; takahara731026@yahoo.co.jp (T.T.);

**Keywords:** atezolizumab/bevacizumab, cabozantinib, hepatocellular carcinoma, liver function, performance status, post-treatment

## Abstract

**Simple Summary:**

The combination of atezolizumab plus bevacizumab (Atz/Bev) is now widely used in clinical practice as a first-line treatment for patients with advanced hepatocellular carcinoma (HCC). However, the established regimen for post-treatment after Atz/Bev is unknown. We investigated the efficacy and safety of cabozantinib in patients previously treated with Atz/Bev in real clinical practice, with a focus on whether patients met criteria of Child–Pugh Class A and Eastern Cooperative Oncology Group performance status (ECOG-PS) score 0/1 at baseline. Our results suggest that cabozantinib in patients with advanced HCC previously treated with Atz/Bev can be expected to yield similar outcomes to those seen in the CELESTIAL trial conducted using cabozantinib for post-sorafenib treatment if patients have good liver function and are in good general condition.

**Abstract:**

(1) Background: This study aimed to investigate clinical outcomes for cabozantinib in clinical practice in patients with advanced hepatocellular carcinoma (HCC) previously treated with atezolizumab plus bevacizumab (Atz/Bev), with a focus on whether patients met criteria of Child–Pugh Class A and Eastern Cooperative Oncology Group performance status (ECOG-PS) score 0/1 at baseline. (2) Methods: Eleven patients (57.9%) met the criteria of both Child–Pugh class A and ECOG-PS score 0/1 (CP-A+PS-0/1 group) and eight patients (42.1%) did not (Non-CP-A+PS-0/1 group); efficacy and safety were retrospectively evaluated. (3) Results: Disease control rate was significantly higher in the CP-A+PS-0/1 group (81.1%) than in the non-CP-A+PS-0/1 group (12.5%). Median progression-free survival, overall survival and duration of cabozantinib treatment were significantly longer in the CP-A+PS-0/1 group (3.9 months, 13.4 months, and 8.3 months, respectively) than in the Non-CP-A+PS-0/1 group (1.2 months, 1.7 months, and 0.8 months, respectively). Median daily dose of cabozantinib was significantly higher in the CP-A+PS-0/1 group (22.9 mg/day) than in the non-CP-A+PS-0/1 group (16.9 mg/day). (4) Conclusions: Cabozantinib in patients previously treated with Atz/Bev has potential therapeutic efficacy and safety if patients have good liver function (Child–Pugh A) and are in good general condition (ECOG-PS 0/1).

## 1. Introduction

Since 2020, based on the positive results of the IMbrave150 trial [1], the combination of atezolizumab plus bevacizumab (Atz/Bev) is now widely used in clinical practice as a first-line treatment for patients with advanced hepatocellular carcinoma (HCC) [2,3,4,5,6,7]. According to clinical-practice guidelines for the treatment of HCC, five molecular targeted agents (MTAs) (sorafenib, regorafenib, lenvatinib, ramucirumab, and cabozantinib) are, similarly, recommended as second-line therapies for patients with advanced HCC once disease progression is seen under treatment with Atz/Bev [8]. However, the IMbrave150 trial did not provide detailed results on treatment after Atz/Bev failure, so no consensus has been reached regarding post-Atz/Bev regimens. Therefore, identifying an effective treatment sequence following Atz/Bev is an important issue.

In the CELESTIAL phase-III clinical trial, cabozantinib, a multi-kinase inhibitor of vascular endothelial growth factor receptor (VEGFR)-1,2,3, growth arrest-specific protein 6 receptor (AXL), and hepatocyte growth factor receptor (MET), was confirmed to be effective and safe as a second- or third-line systemic therapy for patients with advanced HCC following sorafenib [9]. At the time the CELESTIAL trial was conducted, sorafenib was the only first-line systemic agent [10]. However, Atz/Bev has now replaced sorafenib as the standard of care [8,11]. AXL and MET play important roles in HCC progression and resistance to treatment with sorafenib and lenvatinib [12,13,14,15]. VEGFR2, MET, and AXL are involved in immunosuppression of the tumor microenvironment, and cabozantinib given after Atz/Bev inhibits these factors and is expected to have synergistic effects with atezolizumab, which has sustained effects [12,16,17]. There are few reports on the efficacy and safety of cabozantinib in patients previously treated with Atz/Bev [18,19]. The CELESTIAL trial only included patients with both good liver function (Child–Pugh A) and good general condition (Eastern Cooperative Oncology Group performance status (ECOG-PS) 0 or 1). In actual clinical practice, unlike in clinical trials, systemic therapy may need to be administered to patients with poor liver function (Child–Pugh B) or poor general condition (ECOG-PS 2). Little data has been collected on the outcomes of cabozantinib in such patients.

The aim of this study was, therefore, to evaluate the clinical outcomes of cabozantinib in real-world clinical practice among patients with advanced HCC previously treated using Atz/Bev, with a focus on whether these patients met the criteria of Child–Pugh Class A and ECOG-PS score 0/1 at baseline.

## 2. Materials and Methods

### 2.1. Patients

Of the 21 HCC patients introduced to cabozantinib at our institution between February 2021 and October 2022, all 19 patients with a history of Atz/Bev treatment were enrolled in this study to retrospectively evaluate treatment outcomes. All study protocols were approved by the ethics committee at Fujita Health University School of Medicine and were conducted in accordance with the 1975 Declaration of Helsinki. Written informed consent for cabozantinib treatment had been obtained from each patient, but the need for informed consent for participation in this study was waived because of the retrospective design.

### 2.2. Cabozantinib Treatment, Evaluation of Adverse Events and Changes in Liver Function

The recommended starting dose of cabozantinib is 60 mg/day. However, based on initial experience with cabozantinib, some patients were started at a reduced starting dose (40 or 20 mg/day) depending on the condition of the patient. The relative dose intensity (RDI) for Atz/Bev was calculated as the ratio of the dose to the recommended starting dose (60 mg/day). Adverse events (AEs) were evaluated according to Common Terminology Criteria for Adverse Events version 5.0. In the event of a drug-related AE, the dose was reduced or the administration was temporarily suspended until symptoms resolved to grade 1 or 2, according to the guidelines from the manufacturer. Cabozantinib was continued until a potentially fatal AE occurred or clinical tumor progression was observed. Albumin-bilirubin (ALBI) scores [20] were investigated at baseline, week 1, week 2, week 4, and week 6 to assess changes in liver function.

### 2.3. Evaluation of Antitumor Response

Antitumor response was evaluated according to Response Evaluation Criteria in Solid Tumors (RECIST) [21]. Four-phase (unenhanced, late arterial, portal vein, balanced) contrast-enhanced computerized tomography examinations were performed at baseline and 6 weeks after cabozantinib administration, and every 4–10 weeks according to a predetermined schedule.

### 2.4. Statistical Analysis

Statistical analysis was performed using Easy R (EZR) version 1.29 (Saitama Medical Center, Jichi Medical University, Japan) [22]. Progression-free survival (PFS), overall survival (OS), and duration of cabozantinib treatment were evaluated using the Kaplan–Meier method, and differences in survival rates were evaluated by the log-rank test. Factors contributing to PFS or OS were analyzed using multivariate analysis if values of *p* < 0.1 were obtained in univariate analyses. Factors showing values of *p* < 0.05 were interpreted as significant.

## 3. Results

### 3.1. Baseline Characteristics

Baseline characteristics of the 19 HCC patients at the start of cabozantinib are shown in Table 1. Median patient age was 67 years (range, 39–79 years). Fifteen patients (78.9%) were male, 9 (47.4%) had non-viral HCC, 15 (78.9%) had Barcelona Clinic Liver Cancer stage C, and median alpha fetoprotein (AFP) was 885 ng/mL (range, 2.1–625,505 ng/mL). Child–Pugh score was 5 in eight patients, 6 in four patients, 7 in three patients, 8 in two patients, and 9 in two patients. ECOG-PS was 0 in 10 patients (52.6%). Eleven patients (57.9%) showed both Child–Pugh class A and ECOG-PS score 0/1 (CP-A+PS-0/1 group), while the 8 remaining patients (42.1%) did not (Non-CP-A+PS-0/1 group). Cabozantinib was initiated as third-line therapy in nine patients (lenvatinib to Atz/Bev to cabozantinib (n = 7), Atz/Bev to lenvatinib to cabozantinib (n = 2)), fourth-line therapy in seven patients (lenvatinib to Atz/Bev to ramucirumab to cabozantinib (n = 6), Atz/Bev to lenvatinib to ramucirumab to cabozantinib (n = 1)), fifth-line therapy in two patients (lenvatinib to Atz/Bev to ramucirumab to Atz/Bev to cabozantinib (n = 1), Atz/Bev to lenvatinib to ramucirumab to Atz/Bev to cabozantinib (n = 1)), and sixth-line therapy in one patient (sorafenib to regorafenib to lenvatinib to Atz/Bev to ramucirumab to cabozantinib (n = 1)). The initial dose of cabozantinib was 60 mg/day in three patients, 40 mg/day in 12 patients, and 20 mg/day in four patients. The median observation period was 5.1 months (range, 0.5–18.7 months).

### 3.2. Efficacy

The best antitumor responses of cabozantinib according to RECIST in all 19 patients are shown in Table 2. The best antitumor response to cabozantinib alone was complete response (CR) in zero patients, partial response (PR) in one, stable disease (SD) in nine, progressive disease (PD) in two, and not evaluable (NE) in seven. As a result, the objective response rate (ORR) was 5.3% and the disease control rate (DCR) was 52.6%. DCR was significantly higher in the CP-A+PS-0/1 group (81.1%) than in the non-CP-A+PS-0/1 group (12.5%; *p* = 0.006). In terms of the antitumor response to cabozantinib plus additional treatment, the ORR was 10.5% and the DCR was 57.9%, with CR in two patients, PR in zero, SD in nine, PD in one, and NE in seven patients. DCR was also significantly better in the CP-A+PS-0/1 group (90.9%) than in the non-CP-A+PS-0/1 group (12.5%; *p* = 0.001). After the introduction of cabozantinib, two patients (one with BCLC stage B and one with BCLC stage C) in the CP-A+PS-0/1 group were both forced to withdraw cabozantinib due to proteinuria, and received additional treatment with trans-arterial chemoembolization (TACE). In both patients, TACE was performed and SD was obtained, then cabozantinib was resumed, but proteinuria appeared again, making cabozantinib difficult to continue. A patient with BCLC stage B had multiple multinodular HCCs in the left lobe, all of which shrank with cabozantinib (RECIST PR). A patient with BCLC stage C with multiple HCCs in the right lobe and right adrenal metastases achieved mild reduction with cabozantinib (RECIST SD). The two patients subsequently underwent surgical resection (left hepatectomy, right hepatectomy plus right adrenalectomy, respectively) because of their good liver function and general condition, and both patients achieved CR. Of the four HCC patients with BCLC stage B at baseline, three had multiple HCCs (not suitable for TACE) and no additional local treatment was considered.

**Figure 1:**
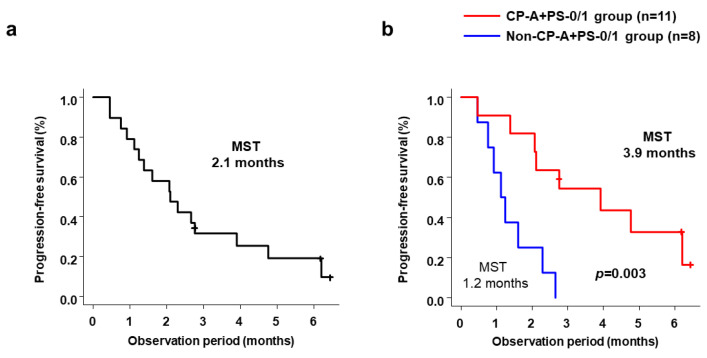
(**a**) Cumulative PFS in all patients and (**b**) cumulative PFS in the CP-A+PS-0/1 and Non-CP-A+PS-0/1 groups. PFS, progression-free survival; CP-A+PS-0/1, Child–Pugh class A and ECOG-PS score 0/1; Non-CP-A+PS-0/1, other than Child–Pugh class A and ECOG-PS score 0/1.

Median PFS for all 19 patients was 2.1 months (range, 1.1–3.9 months) (Figure 1a). Median PFS was significantly longer in the CP-A+PS-0/1 group (3.9 months; range, 1.4–6.2 months) than in the non-CP-A+PS-0/1 group (1.2 months; range, 0.5–2.3 months; *p* = 0.003) (Figure 1b). Table 3 shows baseline factors associated with PFS. Univariate analysis showed that prognostic factors associated with favorable PPS were Child–Pugh Class A and ECOG-PS score 0/1, and age ≥67 years. On multivariate analysis, only Child–Pugh Class A and ECOG-PS score 0/1 were significant independent predictors of favorable PPS (hazard ratio (HR) 0.239; 95% confidence interval (CI) 0.065–0.880; *p* = 0.031).

**Figure 2:**
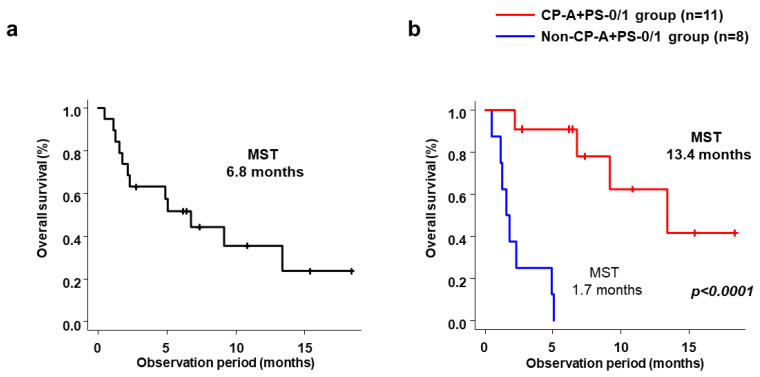
(**a**) Cumulative OS in all patients and (**b**) cumulative OS in the CP-A+PS-0/1 and Non-CP-A+PS-0/1 groups. OS, overall survival; CP-A+PS-0/1, Child–Pugh class A and ECOG-PS score 0/1; Non-CP-A+PS-0/1, other than Child–Pugh class A and ECOG-PS score 0/1.

**Figure 3:**
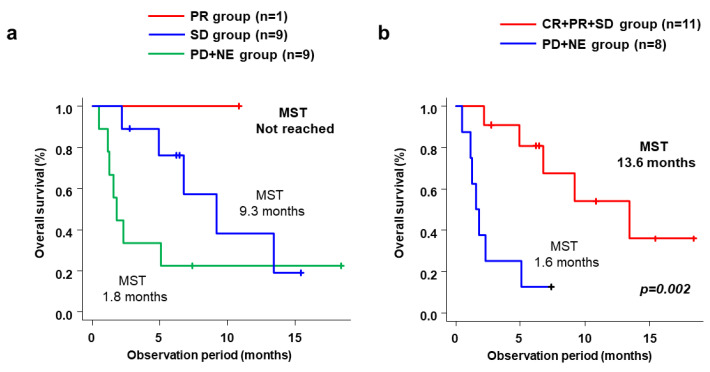
(**a**) Cumulative OS stratified by best antitumor responses to cabozantinib alone based on RECIST in the PR, SD, and PD+NE groups. (**b**) Cumulative OS stratified by best antitumor responses of cabozantinib plus additional treatment based on RECIST in the CR+PR+SD and PD+NE groups. (OS, overall survival; RECIST, Response Evaluation Criteria in Solid Tumors; PR, partial response; SD, stable disease; PD, progressive disease; NE, not evaluable; CR, complete response).

Median OS for all 19 patients was 6.8 months (range, 1.8–13.4 months) (Figure 2a). Median OS was significantly longer in the CP-A+PS-0/1 group (13.4 months; range, 6.8 months–NR) than in the non-CP-A+PS-0/1 group (1.7 months; range, 0.5–4.9 months; *p* < 0.001) (Figure 2b). Table 4 shows the baseline factors associated with OS. In univariate analysis, prognostic factors associated with favorable OS were Child–Pugh class A and ECOG-PS score 0/1, no portal vein tumor thrombosis, and no extrahepatic metastases. Similarly, associated with favorable multivariate analysis were Child–Pugh class A and ECOG-PS score 0/1 (HR 0.043, 95%CI 0.047–0.401; *p* = 0.006), no portal vein tumor thrombosis (HR 0.096, 95%CI 0.014–0.656; *p* = 0.017) and no extrahepatic metastases range (HR 0.055, 95%CI 0.005–0.584; *p* = 0.016). Median OS by best antitumor response with cabozantinib alone was NR (not reached) for PR (one patient), 9.3 months for SD (nine patients), and 1.8 months for PD+NE (Figure 3a). As for best antitumor response with cabozantinib plus additional treatment, median OS was significantly better in the CR+PR+SD group (13.6 months) than in the PD+NE group (1.6 months; *p* = 0.002) (Figure 3b).

### 3.3. Safety

AEs occurring within 6 weeks after cabozantinib initiation are shown in Table 5. AEs of all grades in all 19 patients were, in order of descending frequency, anorexia (52.6%), proteinuria (42.1%), general fatigue (42.1%), and hand-foot syndrome (36.8%). Grade 3 or higher AEs, in order of descending frequency, were proteinuria (31.6%) and general fatigue (10.5%). In the CP-A+PS-0/1 group, proteinuria and hand-foot syndrome were the most common. On the other hand, anorexia and general fatigue were the most common in the non-CP-A+PS-0/1 group.

**Figure 4:**
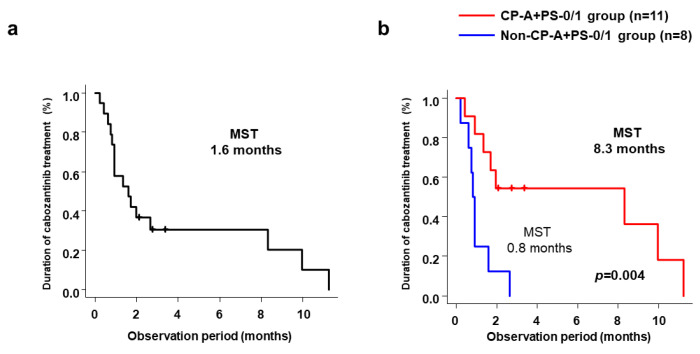
(**a**) Cumulative duration of cabozantinib treatment in all patients. (**b**) Cumulative duration of cabozantinib treatment in the CP-A+PS-0/1 and Non-CP-A+PS-0/1 groups. CP-A+PS-0/1, Child–Pugh class A and ECOG-PS score 0/1; Non-CP-A+PS-0/1, other than Child–Pugh class A and ECOG-PS score 0/1.

Median duration of cabozantinib treatment was 1.6 months (range, 0.8–8.3 months) (Figure 4a). Median duration of cabozantinib treatment was significantly longer in the CP-A+PS-0/1 group (8.3 months; range, 0.9 months–NR) than in the non-CP-A+PS-0/1 group (0.8 months; range, 0.2–1.6 months; *p* = 0.004) (Figure 4b).

The median daily dose of cabozantinib within the 6 weeks after cabozantinib initiation was 19.1 mg/day (range, 6.7–48.6 mg/day) (RDI: 31.8%). Median daily dose of cabozantinib was significantly higher in the CP-A+PS-0/1 group (22.9 mg/day; range, 6.7–48.6 mg/day; RDI, 38.2%) than in the non-CP-A+PS-0/1 group (16.9 mg/day; range, 10.0–21.0 mg/day; RDI, 31.7%; *p* = 0.032).

**Figure 5:**
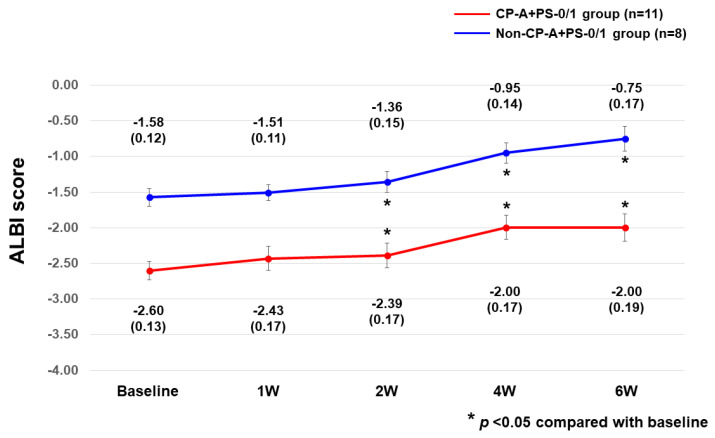
Changes in ALBI scores within 6 weeks after cabozantinib initiation in the CP-A+PS-0/1 and Non-CP-A+PS-0/1 groups. ALBI, albumin–bilirubin; CP-A+PS-0/1, Child–Pugh class A and ECOG-PS score 0/1; Non-CP-A+PS-0/1, other than Child–Pugh class A and ECOG-PS score 0/1; W, week.

The change in ALBI score within 6 weeks was evaluated in all 19 patients. Median (±standard error) ALBI scores at baseline, week 1, week 2, week 4, and week 6 were −1.91 ± 0.15, −1.92 ± 0.16, −1.63 ± 0.17, −1.40 ± 0.17, and −1.62 ± 0.19 (Friedman test, *p* < 0.0001), respectively. In both CP-A+PS-0/1 and non-CP-A+PS-0/1 groups, ALBI scores were significantly worse at 2, 4, and 6 weeks than at baseline (Figure 5).

## 4. Discussion

To the best of our knowledge, this represents the first study to investigate the efficacy and safety of cabozantinib in only patients previously treated with Atz/Bev in real clinical practice. Patients with good liver function (Child–Pugh A) and good general condition (ECOG-PS 0/1) are eligible for clinical trials, but cabozantinib treatment for advanced HCC may, in actual practice, be administered to patients who do not meet these criteria. The present study (19 total patients: Child–Pugh A 63.2%, PS 0/1 84.2%, prior Atz/Bev 100%, cabozantinib as third-line or later treatment in 100%) focused on whether patients met Child–Pugh class A and ECOG-PS score 0/1 at baseline and compared clinical outcomes between groups. Our results showed that efficacy and safety of cabozantinib in patients with Child–Pugh A and ECOG-PS 0/1 (the CP-A+PS-0/1 group) were similar to those in the CELESTIAL study [9], while those without Child–Pugh A and ECOG-PS 0/1 (the Non-CP-A+PS-0/1 group) were poor.

The efficacy of cabozantinib in patients with Child–Pugh A and ECOG-PS 0/1 in clinical trials was reported as follows [9,23,24,25,26,27,28,29]. In the CELESTIAL trial (470 total patients in the cabozantinib group, Child–Pugh A 100%, PS 0/1 100%, prior sorafenib 100%, cabozantinib as third-line or later treatment in 29.6%), ORR was 4%, DCR was 64%, median PFS was 5.2 months, and median OS was 10.2 months in the cabozantinib group [9]. In the Cabozantinib-2003 trial, a phase-II trial in Japanese patients (total 34 patients, 100% Child–Pugh A, 100% PS 0/1, prior sorafenib 58.8%, prior immune checkpoint inhibitor (ICI) 14.7%, cabozantinib as third-line or later treatment in 23.5%), ORR was 0%, DCR was 76.5%, and median PFS was 5.6 months [28,29]. In the present study, the ORR was 9.1%, DCR was 81.1%, median PFS was 3.9 months, and median OS was 13.4 months in the CP-A+PS-0/1 group, results that appeared almost equivalent to those of the CELESTIAL trial and Cabozantinib-2003 trial. Therefore, the results of this study indicate that cabozantinib for patients previously treated with Atz/Bev can be expected to be almost as effective as after sorafenib if patients have good liver function (Child–Pugh A) and are in good general condition (ECOG-PS 0/1).

In real clinical practice, outcomes for cabozantinib including patients other than Child–Pugh A and ECOG-PS 0/1 have been reported [18,19,30,31,32]. Finkelmeier et al. reported that with cabozantinib (total 88 patients, Child–Pugh A 68.2%, PS 0/1 88.6%, prior sorafenib 92%, cabozantinib as third-line or later treatment in 52%), ORR was 7%, DCR was 38.6%, median PFS was 3.4 months, and median OS was 7 months in all patients [30]. PFS was similar in the Child–Pugh A group (3.3 months) and Child–Pugh B group (3.1 months), but OS was significantly longer in the Child–Pugh A group (9.7 months) than in the Child–Pugh B group (3.4 months; *p* = 0.001), and the patients who met CELESTIAL criteria (42%) had a good OS of 11.1 months. Storandt et al. reported outcomes of cabozantinib in patients previously treated with ICI (total 26 patients: Child–Pugh A 72%, prior Ats/Bev 50%, prior nivolumab 46%, prior durvalumab 4%, cabozantinib as third-line or later treatment in 84.6%) [18]. Outcomes for cabozantinib in all patients were: ORR 4%, DCR 27%, median PFS 2.1 months, median OS 7.7 months, and PFS 2.1 months in the Child–Pugh A group and 1.3 months in the Child–Pugh B group, showing no significant difference (*p* = 0.55). These two studies reported no significant difference in PFS between the Child–Pugh A and Child–Pugh B groups. Unlike these reports, in the present study, the non-CP-A+PS-0/1 group was significantly less effective than the CP-A+PS-0/1 group (ORR 0%, DCR 12.5%, median PFS 1.2 months, median OS 1.7 months).

There have been several reports that Child–Pugh B patients have worse OS, PFS, and antitumor response than Child–Pugh A patients due to intolerance of MTA in patients treated with MTA [33,34]. The multinational GIDEON registry study reported that patients with Child–Pugh A had a longer duration of treatment and OS with sorafenib and fewer discontinuations due to AEs than patients with Child–Pugh B [33]. In addition, several studies have shown that baseline ALBI grade is a significant predictor of overall survival and total duration of treatment [35,36,37,38]. Ueshima et al. reported that in lenvatinib therapy, baseline liver function was closely associated with ORR, frequency of AEs, and duration of treatment [35]. A Child–Pugh score of 5 and ALBI grade of 1 predicted a longer duration of lenvatinib treatment and better outcomes.

It is well known that during treatment with MTA, various AEs often necessitate dose reduction or discontinuation [39,40,41]. As a result, the actual dose is often lower than the recommended starting dose. A sub-analysis of the CELESTIAL trial by ALBI grade at baseline was reported, with a median PFS of 6.5 months in the ALBI grade-1 group and 3.7 months in the ALBI grade-2 group [25]. Median RDI of the daily cabozantinib dose was 59.7% and the median duration of cabozantinib treatment was 5.3 months; with ALBI grade 1, the median RDI of cabozantinib daily dose was 61.2% and the median duration of cabozantinib treatment was 4.9 months. With ALBI grade 2, the median RDI of cabozantinib daily dose was 58.8% and median duration of cabozantinib treatment was 3.3 months. In a multivariate analysis, baseline ALBI grade 2 was independently associated with lower OS in the cabozantinib group compared to ALBI grade 1. ALBI-grade-1 patients were more likely to receive subsequent anticancer therapy compared to ALBI-grade-2 patients. In the Cabozantinib-2003 trial [28,29], the median RDI of the daily cabozantinib dose was 35.9% and the median duration of cabozantinib treatment was 5.6 months. According to ALBI grade at the start of cabozantinib, median RDI was the same for ALBI grade 1 (34.6%) and ALBI grade 2 (38.7%), but the median duration of cabozantinib treatment was longer for ALBI grade 1 (7.7 months) than for ALBI grade 2 (5.4 months). In the present study, median RDI for the daily cabozantinib dose was significantly higher in the CP-A+PS-0/1 group (38.2%) than in the non-CP-A+PS-0/1 group (31.7%; *p* = 0.032), and median duration of cabozantinib treatment was significantly longer in the CP-A+PS-0/1 group (8.3 months) than in the non-CP-A+PS-0/1 group (0.8 months; *p* = 0.004). The higher total dose of cabozantinib in the CP-A+PS-0/1 group compared to the non-CP-A+PS-0/1 group is one possible explanation for the favorable outcome in the CP-A+PS-0/1 group.

The major route of excretion of cabozantinib and its metabolites is in the hepatobiliary system [42]. Mild-to-moderate hepatic impairment has been reported to nearly double the exposure to cabozantinib compared to subjects with normal liver function. Therefore, physicians should take into consideration that patients with poor liver function may have significantly lower drug metabolism, higher drug concentrations of cabozantinib, and a higher incidence of AEs. The results of this study showed that the efficacy and tolerability of cabozantinib were significantly poorer in the non-CP-A+PS-0/1 group. Therefore, cabozantinib should not be recommended for Child–Pugh-class-B patients and other alternative treatment options should be chosen if available.

The most frequently occurring AEs in the present study were anorexia, proteinuria, general fatigue, and hand-foot syndrome. AE profiles were the same as those reported for cabozantinib in the CELESTIAL trial [9], the Cabozantinib-2003 trial [28,29], and other real-world clinical reports of cabozantinib [12,13,24,25,26]. In the present study, proteinuria and hand-foot syndrome were the most common in the CP-A+PS-0/1 group. On the other hand, anorexia and general fatigue were the most common in the non-CP-A+PS-0/1 group. AE management in cabozantinib treatment after Atz/Bev is considered similar to that after MTA treatment. For the effective and safe use of cabozantinib, frequent follow-up and early detection and management of AEs (e.g., cabozantinib dose reduction, cabozantinib withdrawal, and symptomatic treatment) are considered important, especially in the initial phase after cabozantinib administration. Among them, anorexia and general fatigue require special attention, as they are AEs that can easily lead to deterioration of liver function [43,44].

Some investigators have reported that starting MTA therapy at lower than recommended doses could be useful in preventing AEs and improving outcomes [19,45]. Tomonari et al. investigated cabozantinib outcomes by starting dose (full- and reduced-dose groups) (total 26 patients, Child–Pugh A 84.6%, ECOG-PS PS 0/1 100%, prior Atz/Bev 80.5%, cabozantinib as third-line or later treatment in 96.2%) [19]. There were no significant differences in ORR, DCR, or PFS between the full-dose group (15 patients) and the dose-reduction group (11 patients). RDI at 4 weeks after starting cabozantinib was 71.1% in the full-dose group and 56.2% in the dose-reduction group, with no significant difference (*p* = 0.13). The incidence of anorexia, fatigue, diarrhea, and discontinuation or dose reduction was significantly higher in the full-dose group. Based on these results, they concluded that starting cabozantinib at a reduced dose may be a safe therapeutic option. Despite the fact that the Cabozantinib-2003 trial was conducted in well-conditioned subjects with Child–Pugh A and ECOG-PS 0/1, the median time to discontinuation due to AEs was 22 days and the overall median RDI for daily doses of cabozantinib was a low 35.9% [28,29]. However, the median PFS was relatively good at 5.6 months. Therefore, in order to minimize the toxicity caused by cabozantinib and maximize the benefit of cabozantinib, even if AEs reduce the dose of cabozantinib and result in a relatively low RDI, the resulting longer duration of cabozantinib treatment may be expected to provide favorable antitumor responses and prolonged prognosis.

In real clinical practice, early deterioration of liver function has been reported after initiation of MTAs such as sorafenib and lenvatinib [35,38]. With regard to changes in ALBI score, the Cabozantinib-2003 trial reported no difference in ALBI change between the ALBI-grade-1 and grade-2 groups, with no significant deterioration in either group compared to baseline [28,29]. However, in this study, ALBI scores were significantly worse than baseline ALBI scores at 2, 4, and 6 weeks in both CP-A+PS-0/1 and Non-CP-A+PS-0/1 groups. One possible reason for the higher incidence of worsening liver function in this study is that in all 19 patients (100%), cabozantinib was initiated as tertiary or later treatment, which may have caused patients to experience a greater deterioration from baseline than if cabozantinib had been used as a second-line treatment.

In 2022, the combination of anti-PD-L1 durvalumab and anti-CTLA 4 tremelimumab showed promising results in the HIMALAYA trial and was positioned as first-line therapy for advanced HCC alongside Atz/Bev [46]. In the future, MTA will likely be administered sequentially after ICI treatment [11]. In particular, cabozantinib will have more opportunities to be used as a third-line or later treatment. The present study population included 100% of patients who used cabozantinib as third-line or later treatment. Our results may provide useful information for future sequential treatment choice. However, comparisons of outcomes with patients who received other therapies or best supportive care are needed to clarify the benefit of using cabozantinib as a third-line or later treatment.

Several limitations to this study need to be recognized. First, the study used a retrospective, nonrandomized design and was conducted at a single center. Second, the study cohorts were heterogeneous in terms of factors affecting efficacy and safety: liver function, cabozantinib treatment line, and cabozantinib initial dose. Third, the sample size was small and the observation period was short for a clinical study. Therefore, additional prospective studies with a larger number of patients in independent cohorts and longer observation periods are needed to validate and confirm the results of this study.

## 5. Conclusions

In conclusion, our present results suggest that cabozantinib, in patients with advanced HCC previously treated with Atz/Bev, has potential therapeutic efficacy and safety if patients have good liver function (Child–Pugh A) and are in good general condition (ECOG-PS 0/1). Further studies are needed to validate and confirm the present findings for cabozantinib in patients previously treated with Atz/Bev.

## Figures and Tables

**Table 1:** Baseline characteristics at initiation of cabozantinib.

Characteristics	n = 19
Median age (years, range)	67 (39–79)
Sex (male/female)	15/4
Etiology (HBV/HCV/non-viral)	6/4/9
Child–Pugh score (5/6/7/8/9)	8/4/3/2/2
ECOG-PS (0/1/2)	10/6/3
Both Child–Pugh class A and ECOG-PS score 0/1 (+/−)	11/8
BCLC stage (B/C)	4/15
HCC size (<50 mm/≥50 mm)	11/8
Number of HCCs (<4/≥4)	1/18
Portal vein tumor thrombosis (−/+)	12/7
Extrahepatic spread (−/+)	8/11
Median serum AFP level (ng/mL, range)	885 (2.1–625,505)
History of hepatic resection (+/−)	10/9
History of radiofrequency ablation (+/−)	3/16
History of trans arterial chemoembolization (+/−)	17/2
History of radiation therapy (+/−)	4/15
History of atezolizumab plus bevacizumab (+/−)	19/0
History of lenvatinib (+/−)	19/0
History of ramucirumab (+/−)	10/9
History of sorafenib (+/−)	1/18
History of regorafenib (+/−)	1/18
Cabozantinib treatment line (3rd-/4th-/5th/6th)	9/7/2/1
Cabozantinib initial dose (60/40/20 mg/day)	3/12/4
Median duration from initial diagnosis of HCC (months)	22.5 (7.9–120.6)
Median observation period (months)	5.1 (0.5–18.7)

HCC, hepatocellular carcinoma; HBV, hepatitis B virus; HCV, hepatitis C virus; ECOG-PS, Eastern Cooperative Oncology Group performance status; BCLC, Barcelona Clinic Liver Cancer; AFP, alpha fetoprotein.

**Table 2:** Best antitumor response to cabozantinib according to RECIST.

	RECIST	All Patients (n = 19)	CP-A+PS-0/1 Group (n = 11)	Non-CP-A+PS-0/1 Group (n = 8)	*p* Value *
Cabozantinib alone	CR/PR/SD/PD/NE, n	0/1/9/2/7	0/1/8/1/1	0/0/1/1/6	
	ORR	5.3%	9.1%	0%	1.000
	DCR	52.6%	81.1%	12.5%	0.006
Cabozantinib + additional treatment	CR/PR/SD/PD/NE, n	2/0/9/1/7	2/0/8/0/1	0/0/1/1/6	
	ORR	10.5%	18.2%	0%	0.485
	DCR	57.9%	90.9%	12.5%	0.001

RECIST, Response Evaluation Criteria in Solid Tumors; CR, complete response; PR, partial response; SD, stable disease; PD, progressive disease; NE, not evaluated; ORR, objective response rate; DCR, disease control rate; CP-A+PS-0/1, Child–Pugh class A and ECOG-PS score 0/1; Non-CP-A+PS-0/1, other than Child–Pugh class A and ECOG-PS score 0/1. * *p* value between CP-A+PS-0/1 and Non-CP-A+PS-0/1 groups.

**Table 3:** Uni- and multivariate analyses of baseline factors associated with good PFS.

	Univariate Analysis		Multivariate Analysis	
Factors	HR (95%CI)	*p* Value	HR (95%CI)	*p* Value
Age (≥67 years)	0.313 (0.099–0.989)	0.048	0.493 (0.144–1.689)	0.260
Sex (male)	0.539 (0.164–1.769)	0.308		
Etiology (HBV or HCV)	0.950 (0.354–3.030)	0.918		
Child–Pugh A and ECOG-PS 0/1 (+)	0.184 (0.054–0.633)	0.007	0.136 (0.035–0.530)	0.031
HCC number (<4)	0.420 (0.051–3.443)	0.419		
HCC size (≥5 cm)	0.874 (0.310–2.463)	0.800		
Vp (−)	0.761 (0.258–2.244)	0.620		
EHS (+)	0.504 (0.174–1.458)	0.206		
AFP level (<400 ng/mL)	0.406 (0.134–1.235)	0.112		

PFS, progression-free survival; HR, hazard ratio; CI, confidence interval; HBV, hepatitis B virus; HCV, hepatitis C virus; ECOG-PS, Eastern Cooperative Oncology Group performance status; HCC, hepatocellular carcinoma; Vp, portal vein tumor thrombosis; EHS, extrahepatic spread; AFP, alpha-fetoprotein.

**Table 4:** Uni- and multivariate analyses of baseline factors associated with good OS.

	Univariate Analysis		Multivariate Analysis	
Factors	HR (95%CI)	*p* Value	HR (95%CI)	*p* Value
Age (≥67 years)	0.634 (0.200–2.013)	0.440		
Sex (female)	0.901 (0.241–3.368)	0.877		
Etiology (HBV or HCV)	0.527 (0.167–1.669)	0.276		
Child–Pugh A and ECOG-PS 0/1 (+)	0.039 (0.005–0.324)	0.003	0.044 (0.005–0.401)	0.005
HCC number (≥4)	0.976 (0.127–7.806)	0.981		
HCC size (<5 cm)	0.524 (0.155–1.770)	0.298		
Vp (−)	0.296 (0.078–1.118)	0.072	0.096 (0.014–0.656)	0.017
EHS (−)	0.226 (0.048–.053)	0.058	0.055 (0.005–0.584)	0.016
AFP level (<400 ng/mL)	0.417 (0.123–1.413)	0.160		

OS, overall survival; HR, hazard ratio; CI, confidence interval; HBV, hepatitis B virus; HCV, hepatitis C virus; ECOG-PS, Eastern Cooperative Oncology Group performance status; HCC, hepatocellular carcinoma; Vp, portal vein tumor thrombosis; EHS, extrahepatic spread; AFP, alpha-fetoprotein.

**Table 5:** Adverse events within 6 weeks of cabozantinib administration (n = 19).

	All Patients (n = 19)		CP-A+PS-0/1 Group (n = 11)		Non-CP-A+PS-0/1 Group (n = 8)	
	Any Grade n, (%)	Grade ≥3 n, (%)	Any Grade n, (%)	Grade ≥3 n, (%)	Any Grade n, (%)	Grade ≥3 n, (%)
Anorexia	10 (52.6)	1 (5.2)	5 (45.5)	0	5 (62.5)	1 (12.5)
Proteinuria	8 (42.1)	6 (31.6)	6 (54.5)	5 (45.5)	2 (25.0)	1 (12.5)
General fatigue	8 (42.1)	2 (10.5)	4 (36.4)	0	4 (50.0)	2 (25.0)
Hand-foot syndrome	7 (36.8)	1 (5.3)	6 (54.5)	1 (9.1)	1 (12.5)	0
Diarrhea	6 (31.6)	1 (5.3)	5 (45.5)	0	1 (12.5)	1 (12.5)
Hypothyroidism	6 (31.6)	0	3 (27.3)	0	3 (37.5)	0
Bleeding	3 (15.8)	1 (5.3)	1 (9.1)	0	2 (25.0)	1 (12.5)
Hypertension	3 (15.8)	0	2 (18.2)	0	1 (12.5)	0
Fever	3 (15.8)	0	2 (18.2)	0	1 (12.5)	0

CP-A+PS-0/1, Child–Pugh class A and ECOG-PS score 0/1; Non-CP-A+PS-0/1, other than Child–Pugh class A and ECOG-PS score 0/1.

## Data Availability

The data presented in this study are available on request from the corresponding author. The data are not publicly available due to institutional restrictions.
